# Assessment of the Multiple Sclerosis Severity Score and the Age-Related Multiple Sclerosis Severity Score as health indicators in a population-based cohort

**DOI:** 10.1007/s10072-024-07767-3

**Published:** 2024-09-17

**Authors:** Laura Bau, Elisabet Matas, Lucía Romero-Pinel, Isabel León, Albert Muñoz-Vendrell, Pablo Arroyo-Pereiro, Antonio Martínez-Yélamos, Sergio Martínez-Yélamos

**Affiliations:** 1 Multiple Sclerosis Unit, Department of Neurology, Hospital Universitari de Bellvitge, L’Hospitalet de Llobregat, Barcelona, Spain; 2 Neurologic Diseases and Neurogenetics Group, Neuroscience Program, Institut d’Investigació Biomèdica de Bellvitge (IDIBELL), L’Hospitalet de Llobregat, Barcelona, Spain; 3 Departament de Ciències Clíniques, Facultat de Medicina, Universitat de Barcelona, Barcelona, Spain

**Keywords:** Multiple sclerosis, Disability, MSSS, ARMSSS, Health outcome measures

## Abstract

**Background:**

People with multiple sclerosis (MS) present varying degrees of disability throughout their disease course. The Multiple Sclerosis Severity Score (MSSS) and the Age-Related Multiple Sclerosis Severity Score (ARMSSS) adjust the Expanded Disability Status Scale (EDSS) according to disease duration and age, respectively. These measures could be useful for quantifying MS severity and as health outcome indicators for benchmarking in population-based settings. The aim of this study was to describe the severity of MS in our health district using the MSSS and ARMSSS and to assess their consistency over time.

**Methods:**

This population-based study included patients from our health district who were diagnosed with MS according to the 2010 McDonald criteria, had a disease duration of at least one year and were followed up in our MS unit. Sex, age at onset, disease duration, clinical course, age and irreversible EDSS at the last follow-up visit were collected, and the MSSS and ARMSSS were calculated at two time points: 2017 and 2020.

**Results:**

One hundred seventy-seven patients were included in 2017, and 208 in 2020. The prevalence of MS was 90 and 104 per 100,000 inhabitants, respectively. The median MSSS was 1.77 (IQR 0.76–4.28) in 2017 and 2.03 (IQR 0.82–4.36) in 2020. The median ARMSSS was 2.90 (IQR 1.47–5.72) in 2017 and 2.93 (IQR 1.51–5.56) in 2020. No significant differences were found.

**Conclusions:**

According to the MSSS and ARMSSS, the severity of MS in our area is mild, and these instruments are consistent. These measures could be reliable health outcome measures.

## Introduction

Multiple sclerosis (MS) is the most common inflammatory disease of the central nervous system. The clinical course is heterogeneous, and people with MS (pwMS) show varying degrees of disability during the course of the disease [1]. The Expanded Disability Status Scale (EDSS) is the most commonly used instrument to assess neurological impairment in MS patients in clinical practice. The EDSS is based on the neurological examination and scores range from 0 (normal exam) to 10 (death due to MS) in intervals of 0.5 [2]. The EDSS is a validated scale that is widely used in MS clinical trials, but it has some limitations, such as poor reproducibility, low sensitivity to change, and underestimation of some symptoms, such as impaired cognitive status and impairment in the upper extremities [3].

Other measures have been developed to determine MS severity using a single assessment. The Multiple Sclerosis Severity Score (MSSS) is based on the EDSS score standardized by disease duration. The resulting score ranges from 0 to 10, with 5 as the median, and indicates the severity of the disease in a given patient relative to other patients with similar disease durations. This scale is applicable to patients with a disease duration of at least one year. The MSSS was developed from a large dataset including 9,892 patients from 11 countries and is a useful tool for comparing groups of patients [4, 5]. Similarly, the Age-Related Multiple Sclerosis Severity Score (ARMSSS) adjusts the EDSS score according to age [6]. For the ARMSSS, an MS patient’s disease severity is compared to that of other MS patients of similar age in a single assessment using the EDSS score and age. As disease duration is not needed for score calculation, the ARMSSS addresses the difficulty of retrospectively determining disease onset in clinical practice. In addition, by allowing comparison by age, environmental factors, comorbidities and any age-related conditions are already standardized [6].

The primary use of the MSSS is to describe the disease severity of groups of patients, allowing comparisons between groups or stratification for trials, or as a descriptor for the general population [4, 7]. Although the MSSS has been used to predict outcomes for individuals, its use is controversial due to potential fluctuations over time that make it difficult to apply at the individual level rather than the group level [7–10]. Therefore, these instruments could be useful for quantifying disease severity and as indicators of health outcomes in the population. Efforts have been made to define and evaluate sets of quality indicators to monitor MS outcomes in the real world [11–14]. The availability of these measuring instruments allows for monitoring and benchmarking of outcomes, as well as improving outcomes by learning from different clinical practices, identifying gaps in clinical care and informing patients of the results [11–14]. The aim of this study was to describe the severity of MS in our health district using the MSSS and the ARMSSS and to assess their consistency over time.

## Methods

### Study design and participants

An observational study was performed in the Multiple Sclerosis Unit of Hospital Universitari de Bellvitge. This MS unit, which is located in southern Barcelona in Catalonia, a region in northeastern Spain, is the only centre for demyelinating diseases in our health district. Patients who were diagnosed with MS according to the 2010 McDonald criteria [15], had a disease duration of at least one year, were followed up in our MS unit within the previous 18 months and were from our health district were selected. Patient selection was performed in April 2017 and May 2020 to compare our population at two similar time points. Some patients met the inclusion criteria in both analyses. Patients from other health districts who were followed up at our centre were not included.

The Clinical Research Ethics Committee of Hospital Universitari de Bellvitge approved the study. All the patients who participated in the study signed an informed consent form, and the data were collected anonymously. The study was performed in accordance with relevant guidelines and regulations.

### Outcome measures

The severity of MS according to the MSSS and ARMSSS was studied at two different time points: 2017 and 2020. Study variables were collected at the last follow-up visit of each patient and included sex, age at MS onset, disease duration, clinical course of the disease, age and irreversible EDSS score (defined as the EDSS score confirmed after 6 months). Clinical data were collected prospectively in the European Database for Multiple Sclerosis (EDMUS) during routine follow-up visits at least every six months and at the time of relapse [16]. The EDSS score was determined by a certified neurologist (Neurostatus) [3]. The MSSS and ARMSSS were determined using a Stata software package created by the developers of the ARMSSS [6].

### Statistical analysis

A descriptive analysis was performed. Frequencies for qualitative variables and means and standard deviations or medians and interquartile ranges for quantitative variables were calculated. Clinical characteristics and disease severity measures from the 2017 and 2020 cohorts were compared using the chi-square test and Mann‒Whitney U test, as appropriate. All tests were conducted at a significance level of 5%. Statistical analysis was performed with Stata (StataCorp. 2013. Stata: Release 13. Statistical Software. College Station, TX: StataCorp LP).

## Results

One hundred eighty-one patients from our health district were diagnosed with MS and followed up in our MS unit in 2017. Considering that our centre was the referral hospital for 201,192 inhabitants in 2016, the calculated prevalence of MS in our health district was 90 cases per 100,000 inhabitants. In 2020, 213 patients were followed up in our MS unit. Based on a population of 206,040 inhabitants in 2019, the prevalence was 104 cases per 100,000 inhabitants.

According to the inclusion criteria for this study, 177 patients in April 2017 and 208 patients in May 2020 were eligible and included. The clinical characteristics of the patients did not significantly differ between 2017 and 2020, as shown in Table 1. Most of the patients had a relapsing course of the disease; women accounted for two-thirds of the sample, the mean age at disease onset was approximately 32 years, and the median EDSS score was 2.0. No differences were found in the proportion of patients on disease-modifying treatment (DMT).

**Table 1:** Patient clinical characteristics

	2017	2020	*p*
***n***	177	208	
**Mean age at data collection (years)** (SD)	49.8 (13.3)	50.7 (13.3)	0.504
**Female** (%)	71.2	68.3	0.535
**Mean age at onset of MS (years)** (SD)	31.7 (11.3)	32.7 (11.6)	0.426
**Mean disease duration (years)** (SD)	17.5 (10.6)	17.5 (11.7)	0.725
**Median EDSS** [IQR]	2 [1.5–3.5]	2 [1.5–3.5]	0.655
**Clinical course** (%)			0.837
RRMS	87.6	85.6	
SPMS	7.3	8.2	
PPMS	5.1	6.2	
**Disease modifying treatment** (%)	63.3	67.3	0.407

*EDSS: Expanded Disability Status Scale. PPMS: Primary progressive multiple sclerosis. RRMS: Relapsing–remitting multiple sclerosis. SPMS: Secondary progressive multiple sclerosis*

**Fig. 1:**
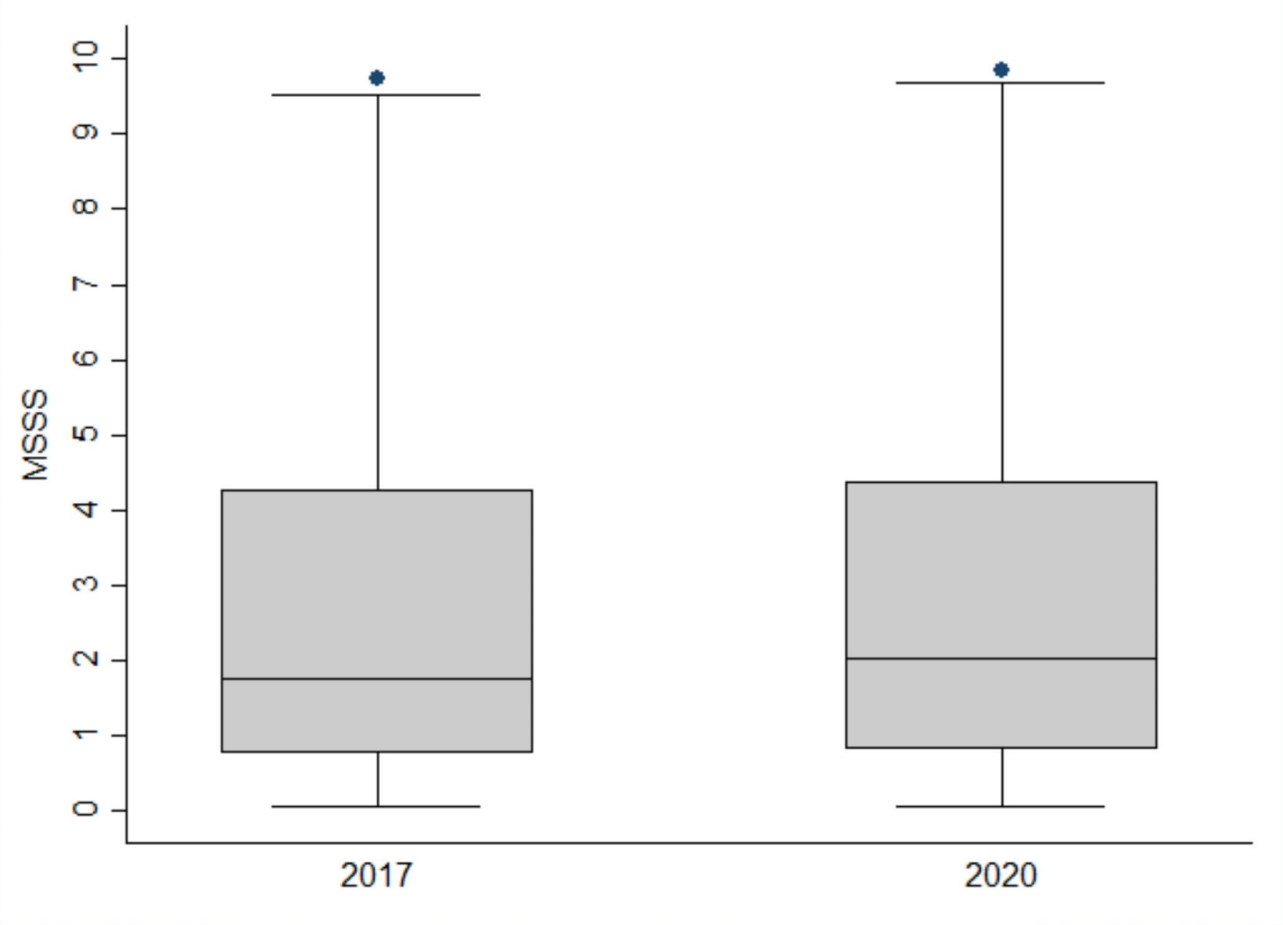
Box plot of the distribution of MSSS in 2017 and 2020

**Fig. 2:**
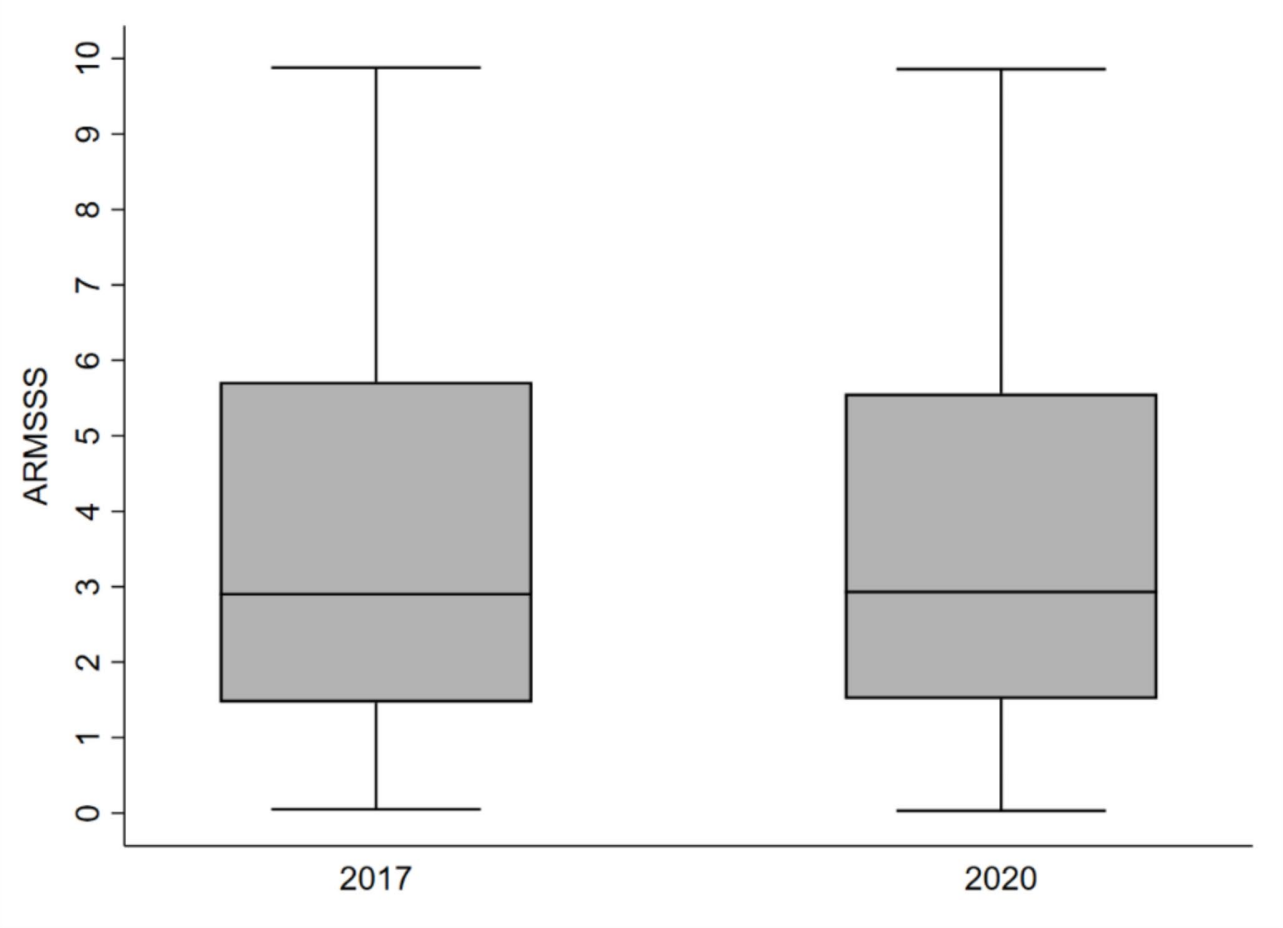
Box plot of the distribution of ARMSSS in 2017 and 2020

The median MSSS was 1.77 (IQR 0.76–4.28) in 2017 and 2.03 (IQR 0.82–4.36) in 2020. The median ARMSSS was 2.90 (IQR 1.47–5.72) in 2017 and 2.93 (IQR 1.51–5.56) in 2020. The distributions of the MSSS and ARMSSS at the analysed data collection times are shown in Figs. 1 and 2, respectively. The median MSSS and ARMSSS at both data collection times did not significantly differ (*p* = 0.504 and *p* = 0.909, respectively).

## Discussion

In this study, we analysed MS severity in our health district according to the MSSS and ARMSSS and assessed consistency at two different time points. Our results suggest that these instruments can be used as health outcome indicators. Such measures are important for decision-making and improving the quality of care in a population-based setting.

The prevalence of MS in our health district was in line with the expected prevalence. According to previous studies, the estimated crude prevalence of MS among Spanish-born individuals in our region was 79.9 cases (95% CI: 66.3–95.6) per 100,000 inhabitants and 91.2 cases (95% CI: 75.5–109.2) per 100,000 inhabitants [17]. Similarly, a recent study revealed a nonadjusted prevalence of MS of 111.9 cases (95% CI: 87.7–142.9) per 100,000 inhabitants in a region in southeastern Spain [18]. As the prevalence observed in this study was within the expected range for our geographical area, we assumed that most of the pwMS in our health district were followed up in our centre; thus, the probability of ascertainment bias was minimized. Patients referred from other health districts for follow-up at our centre were not included in the study. Therefore, although this was a single-centre study, the described cohort can be considered a population-based cohort.

The median MSSS and ARMSSS values of approximately 1.9 and 2.9, respectively, obtained in our cohort indicated mild disease severity. With respect to standardized measures, when comparing patients with the same disease duration or age, patients with values below 5 (the median) have a more favourable outcome. Importantly, the results were consistent in both study years. These measures were unlikely to change substantially when estimated over a short time interval since most patients were likely to be in both groups, and disease management, including diagnostic procedures, available treatments, and health policies, remained stable. The absence of significant changes may indicate that the measures are stable and not affected by irrelevant factors. Therefore, if a significant change is identified, it would be important to investigate the underlying causes.

To date, few studies in the literature have assessed disease severity in a population using the MSSS. A multicentre study performed in Morocco obtained a median MSSS value of 6.39 (IQR 3.17–8.83) in a sample of 460 patients. In contrast with our results, 17.9% of the patients presented with primary progressive MS, while 61.9% presented with relapsing-remitting MS. It was concluded that this cohort had more malignant forms of the disease and a greater frequency of rapid progressors (MSSS > 5), a finding consistent with the more aggressive disease course that has been described in studies of North African migrants to Europe [19]. Another study performed in Kuwait aimed to assess the influence of lifestyle factors on MS severity measured with the MSSS [20]. In this cohort of 128 patients, the mean MSSS was 4.07 (SD 2.29). Most of these patients presented with relapsing-remitting MS (96.9%) and had EDSS scores between 0 and 3 (81.3%), indicating mild disease severity. Notably, the median disease duration was 3 years (IQR 2–8).

These instruments may be useful for comparing populations or clinical settings [4]. For instance, a study revealed differences between MS patients from the United Kingdom (UK) and Japan using the MSSS [21]: the median value in the cohort of UK patients was 5.9 (IQR 2.6–7.9), compared to that of 3.3 (IQR 1.8–5.7) in the cohort of Japanese patients. These differences may be explained by several factors. First, patients with primary progressive MS accounted for 12.9% of the patients in the UK cohort, while patients with this disease course accounted for 3% of the patients in the Japanese cohort. However, statistically significant differences persisted when only patients with relapsing-onset forms were analysed. Additionally, the percentage of treated patients was greater in the Japan cohort than in the UK cohort, but the authors considered that exposure to treatment in this study did not affect the disability score [21].

In addition, the MSSS has been used in clinical settings or geographic areas to compare disease severity among groups of patients with different characteristics, ethnicities or countries of origin [22, 23]. This score has also been widely used to study factors associated with disability in MS patients [24–26].

Previous studies were mostly hospital-based studies conducted at referral centres where patients with an aggressive disease course may be overrepresented. In population-based studies, all MS patients are included, even those with benign forms of the disease; therefore, a more favourable course of MS is evidenced in population-based studies than in hospital or clinic-based cohort studies [27]. These differences in patient selection, conditioned by the study setting, may account for the milder disease severity observed in our study.

The differences in the characteristics between our cohort and the original cohort in which the MSSS was described may partially account for the obtained results. Our cohort had a lower proportion of patients with progressive disease, which could influence the outcome, as disability progression differs in primary progressive and relapsing-onset MS patients [28]. Additionally, in the original MSSS cohort, some patients’ EDSS scores were obtained during disease relapse, whereas in our study, irreversible EDSS scores were used to calculate the MSSS and ARMSSS, excluding the EDSS score obtained during relapse.

Importantly, other recent studies have also revealed a trend towards a less severe course of MS [29, 30]. For instance, a study from the New York State MS Consortium cohort revealed a lower MSSS in more recent participants than in earlier participants, independent of age, sex, ethnicity and time to diagnosis. The authors concluded that this reduction in disease severity may be explained by DMT exposure, as the proportion of participants who received DMT increased from 51 to 71% during the interval studied [31]. Similarly, the EPIC study cohort, a single-centre prospective MS cohort in the treatment era, showed significantly lower rates of worsening and progression to secondary progressive MS than did previous natural history studies. The median MSSS was 2.4 (IQR 0.9–4.3), with a median follow-up time of 9.8 years (IQR 8.6–10.2); 4% of the patients had primary progressive MS, and 61.1% of the patients received treatment [32]. These data are very similar to those obtained in this study. A greater percentage of patients in both studies received DMT than in the original MSSS cohort, where only 27% of patients received treatment [4]. A recent study examined the long-term effects of DMT exposure on disability and quality of life in two cohorts from Australia and New Zealand with different DMT subsidy policies. The results showed a significantly greater median MSSS (3.79 ± 4.02 vs. 3.05 ± 3.45) in the New Zealand cohort, where 50.4% of the participants had DMT exposure, than in the Australian cohort, where 93.9% of the participants were receiving DMT. Additionally, the study revealed a significantly greater quality of life in the Australian cohort [33].

The MSSS and ARMSSS are easily calculated from a single EDSS assessment, which is widely used in clinical practice, and disease duration or age, respectively; therefore, these measures have been used in studies to assess factors associated with disability in MS patients and have allowed for comparisons not only between different countries but also between different cohorts in the same geographical area. In a population setting, these instruments can be explored as health outcome indicators.

Health outcome indicators are useful for monitoring changes in a population or comparing health systems. Usually, clinical disease activity outcomes such as the annualized relapse rate or time to confirmed disability worsening according to the EDSS are studied in MS [34, 35]. However, when comparing MS populations, the use of the MSSS and ARMSSS as health outcome indicators would be useful for benchmarking. In a population, an increase in the median MSSS and ARMSSS could indicate a worsening of the quality of care provided by a health care provider to patients with MS in the geographical area evaluated. On the other hand, if two different populations whose health care providers have different strategies for the management of MS have very different median MSSS and ARMSSS values, this could help to identify more effective strategies or interventions. Treatment strategies for MS are not limited to the prescription of certain DMT. They also include factors such as early diagnosis, early treatment or the appropriate use of DMT. Treatment strategies could also include interventions for general health factors that may influence long-term disability in pwMS, such as smoking cessation, the prevention of obesity and the promotion of regular physical exercise [20, 36]. The accessibility of health services and physical and cognitive therapy could be factors that influence long-term disability in pwMS. Thus, healthcare providers who adopt a more comprehensive approach to the management of MS should expect to observe a lower disability in their patient populations in terms of the MSSS and ARMSSS.

This study has several limitations, such as the short interval between measurements. Although this can be considered a limitation, it was necessary to ensure the consistency of the indicators, as changes are not to be expected in such a short period. The ability to detect changes when there are modifications in disease management or technological innovations remains to be evaluated. Another limitation could be that treatment exposure has not been studied in detail. Although the percentage of patients who received DMT at each time point was analysed, the effects of exposure to different DMTs, the treatment strategy used or the treatment duration on the MSSS and ARMSSS were not studied.

## Conclusion

In conclusion, the results of this study indicate that disease severity in our area is mild according to the MSSS and ARMSSS, and these instruments seem to be consistent. These scores are easy to assess and could be used as reliable health outcome measures for comparing disease severity between cohorts or monitoring a cohort over time. Further research is needed to obtain more insight into the usefulness of these scores for decision making in healthcare management in a population-based setting.

## Data Availability

Supporting data can be obtained from the corresponding author upon reasonable request.
